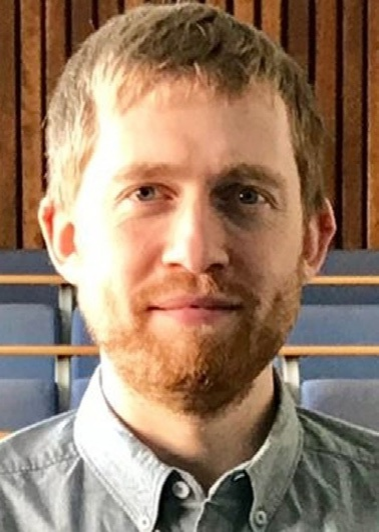# As Nice as π: Aromatic Reactions Activated by π‐Coordination to Transition Metals

**DOI:** 10.1002/chem.202004621

**Published:** 2021-01-07

**Authors:** Luke J. Williams, Yunas Bhonoah, Luke A. Wilkinson, James W. Walton

**Affiliations:** ^1^ Department of Chemistry Durham University South Road Durham DH1 3LE UK; ^2^ Syngenta Jealott's Hill International Research Centre Bracknell Berkshire RG42 6EY UK; ^3^ Department of Chemistry University of York Heslington York YO10 5DD UK

**Keywords:** arene complexes, catalysis, coordination complexes, transition metals

## Abstract

π‐Coordination of aromatic molecules to metals dramatically alters their reactivity. For example, coordinated carbons become more electrophilic and C−H bonds of coordinated rings become more acidic. For many years, this change in reactivity has been used to trigger reactions that would not take place for uncoordinated arenes, however, there has been a recent resurgence in use of this technique, in part due to the development of catalytic reactions in which π‐coordination is transient. In this Minireview, we describe the key reaction chemistry of arenes coordinated to a range of transition metals, including stereoselective reactions and industrially relevant syntheses. We also summarise outstanding examples of catalytic processes. Finally, we give perspectives on the future direction of the field, with respect to both reactions that are stoichiometric in activating metals and those employing catalytic metal.

## Graphical abstract

**Easy as π**: π‐Coordination of aromatic compounds to transition metals dramatically alters the reactivity of the arene. In this Minireview, the authors highlight reactions that exploit this increased activity, selecting examples that are both stoichiometric and catalytic in the activating metal. The advantages and limitations of the activation strategy are discussed and it is considered which areas of future research will benefit from this technique.

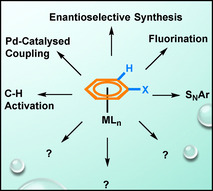

## Introduction

**Figure 1:**
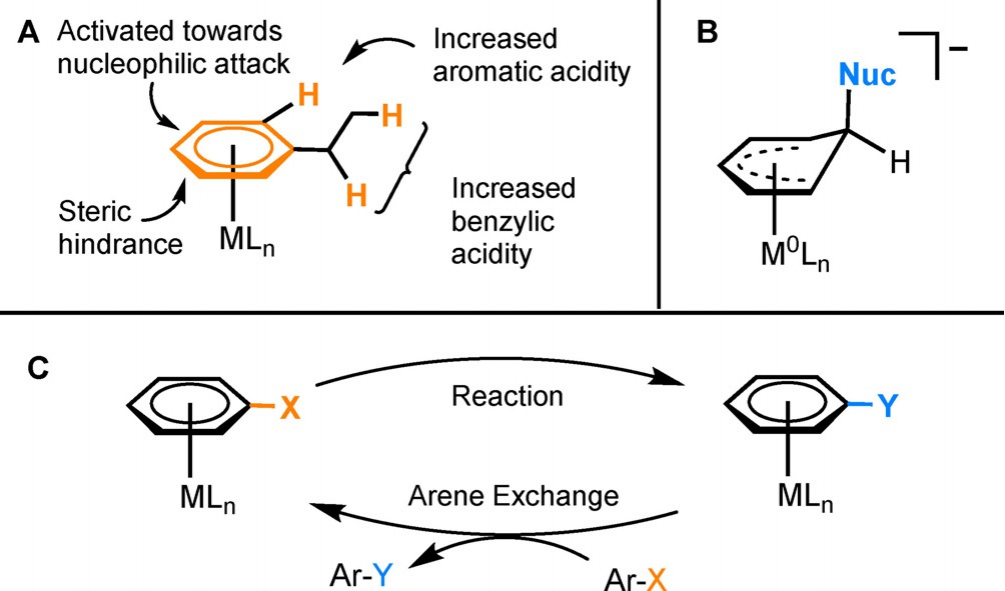
A) Enhancement of arene reactivity, caused by η^6^‐complexation to a metal centre. B) A Meisenheimer complex. C) General mechanism of an aromatic transformation catalysed by η^6^‐coordination to a metal fragment.

Since their discovery in the 1950s by E. O. Fischer,^[1]^ η^6^‐arene complexes of transition metals have been of widespread interest, due to the enhancement of the reactivity of the arene upon complexation (Figure 1 A). Coordination allows for the aromatic compound to undergo many reactions that are not possible for the corresponding unbound arene.^[2]^ For example, nucleophilic attack at the coordinated ring can result in substitution reactions of ring‐bound substituents or, where substitution is not favoured, the formation of η^5^‐coordinated Meisenheimer complexes (Figure 1 B), which are often stable and isolable. Other reactions at the bound aromatic include deprotonation, with subsequent reaction with electrophiles, and oxidative addition. Following aromatic transformations, arenes are typically liberated from the metal centre by photolytic or thermolytic techniques.^[3]^ For some arenes and transition metals, π‐complexation is a reversible process. Hence, it is possible to adapt the stoichiometric transformation of a coordinated arene complex into a process catalytic, with respect to the ML_n_ core (Figure 1 C). This attractive prospect requires balance between the reactivity of the coordinated arene and exchange of the coordinated arene product with the next equivalent of reactant arene.

In this Minireview, we highlight the key advances over the last 20 years in both reactions of η^6^‐coordinated aromatics and in reactions catalytic in the activating metal. We restrict our scope to C_6_ aromatics and omit examples that include chromium complexes as the activating group, which have been well reviewed elsewhere by Kündig^[4]^ and by Matsuzaka and Takemoto.^[5]^ We pay particular focus to suggestions for future development in the field and pick out key publications that will pave the way for new reactions.

## Reactivity of π‐arene Transition Metal Complexes

### Chromium complexes

The early work on π‐arene transition metal complexes focussed on [(η^6^‐C_6_H_6_)Cr(CO)_3_] and related complexes to develop the fundamental understanding of the reactivity of the coordinated arene. The reader is guided to several excellent reviews and book chapters on the early understanding of this complex and its derivatives.[^4^, ^6^] More recently, the reactivity of these Cr species has further developed into C−H activation and a range of Pd‐catalysed coupling processes, which have also been reviewed.[^5^, ^7^]

### Molybdenum complexes

**Scheme 1:**
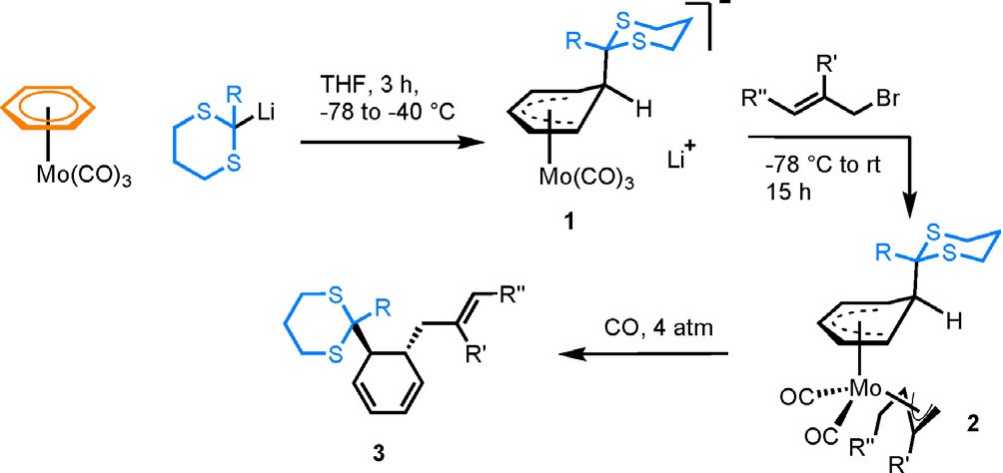
Diastereoselective synthesis of a 1,2‐disubstituted cyclohexadiene from [(η^6^‐C_6_H_6_)Mo(CO)_3_].^[8]^

In comparison with chromium, its Group 6 congener molybdenum has received far less attention in the synthesis and application of π‐arene complexes. This is likely due to the lower kinetic stability of the Mo complexes, limiting their practical utility. In a rare example, the complex [(η^6^‐C_6_H_6_)Mo(CO)_3_] was treated with an alkyllithium nucleophile, resulting in formation of an anionic Meisenheimer complex **1** (Scheme 1).^[8]^ Further treatment with allyl bromide yielded the neutral complex [(η^3^‐allyl)(η^5^‐cyclohexadienyl)Mo(CO)_2_] (**2**), which, on treatment with CO gas, reductively eliminated to give exclusively one diastereomer of a 1,2‐disubstituted cyclohexadiene **3**. This serves as an elegant example of the stereocontrol imparted by the metal complex, with the initial nucleophilic attack occurring at the top face of the arene. Further examples of Mo complex reactivity are rare, but sandwich complexes of the formula [Mo(η^6^‐arene)_2_] have been shown to be active towards lithiation, facilitating addition of electrophiles to the arene ring^[9]^ and complexes of the form [Mo(η^6^‐arene)(PR_3_)_3_] can be used as precursors to other heteroleptic complexes, due to the lability of the coordinated arene.^[10]^

### Manganese complexes

**Figure 2:**
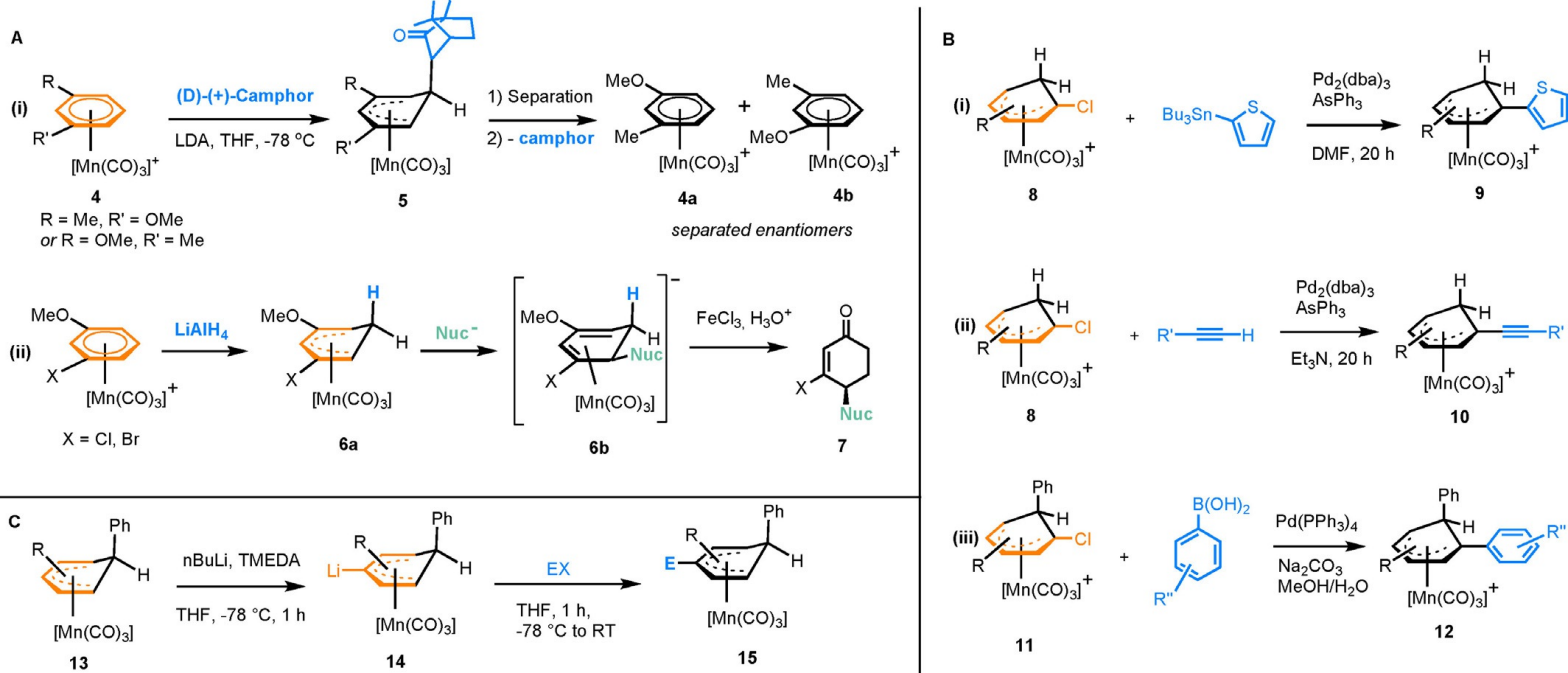
A) Resolution of Mn^I^ π‐arene complexes with planar chirality and subsequent conversion into substituted cyclohexenones. B) Stille, Sonogashira and Suzuki–Miyaura coupling reactions of Mn^I^ Meisenheimer complexes. C) Lithiation of the complex [(η^5^‐cyclohexadienyl)Mn(CO)_3_] followed by electrophilic quenching (X=halide).

π‐Arene complexes of the [Mn(CO)_3_]^+^ fragment are extremely stable and, due to the net positive charge, are highly susceptible to nucleophilic attack at the arene ring. Rose et al. used the [(η^6^‐arene)Mn(CO)_3_]^+^ framework to develop enantioselective syntheses of substituted cyclohexenones from *meta*‐halogenoanisoles.^[11]^ Coordination of 1,3‐disubstitued benzene to [Mn(CO)_3_]^+^ gives a pair of complexes (**4**) with planar chirality (Figure 2 A). Reaction with enantiopure (D)‐(+)‐camphor forms a pair of diastereomeric Meisenheimer complexes (**5**), which can be separated by chromatography. The chiral auxiliary can then be removed to yield enantiopure complexes **4 a** and **4 b**. As shown in Figure 2 A, these complexes can be converted into enantiopure cyclohexenones in three steps. In a related study, the natural products stemofurans were synthesised by nucleophilic substitution of hydrogen in [(η^6^‐arene)Mn(CO)_3_]^+^ complexes with benzofuran.^[12]^ In another study, the scope of reactivity of Meisenheimer complexes structurally related to **6 a** towards various nucleophiles was established,^[13]^ while earlier developments have been reviewed in detail by Rose and Rose‐Munch.^[14]^ Meisenheimer complexes of Mn^I^ are often active towards organometallic coupling reactions. For example, a series of aryl chloride derived complexes (**8**), undergo Stille and Sonogashira couplings to give complexes **9** and **10**, respectively (Figure 2 B).^[15]^ A more recent study reported the Suzuki–Miyaura coupling of similar chloro‐substituted η^5^‐coordinated Meisenheimer complexes (**11**), to give the arylated product **12**.^[16]^ Rearomatisation of the cyclohexadienyl ring in **12** can be achieved with the mild oxidant, trityl chloride. Further examples of Pd‐catalysed reactions^[17]^ include Sonogashira coupling to facilitate formation of a dinuclear Mn‐Fe complex, which has potential electronic applications^[18]^ and the synthesis of organometallic phosphine complexes, used as ligands in catalytic allylations.^[19]^ Further owing to their high stability, Meisenheimer complexes of Mn^I^ are active towards lithiation, enabling the installation of electrophilic functional groups to the ring before rearomatisation.^[20]^ Optimisation of the lithiation/electrophilic quenching procedure led to a model system (Figure 2 C), in which the reactions occur *para* to the sp^3^ tetrahedral carbon of the Meisenheimer ring (**13**), forming complex **15** after electrophilic quench, which could be purified to diastereomeric excesses of >85 %. Reactivity of Meisenheimer Mn complexes has also been exploited to form keto‐[^21^, ^22^] and alcohol‐^[23]^ substituted cyclohexadienyl complexes. In another study, Grignard reagents were reacted with keto‐substituted cyclohexadienyl, giving the corresponding alcohols in excellent yields.^[24]^ Acidification of these alcohols resulted in dehydration reactions, giving rise to a series of novel η^5^‐cyclohexadienyl complexes substituted by a C=C double bond conjugated to the π‐system of the ring.

### Technetium and rhenium complexes

**Figure 3:**
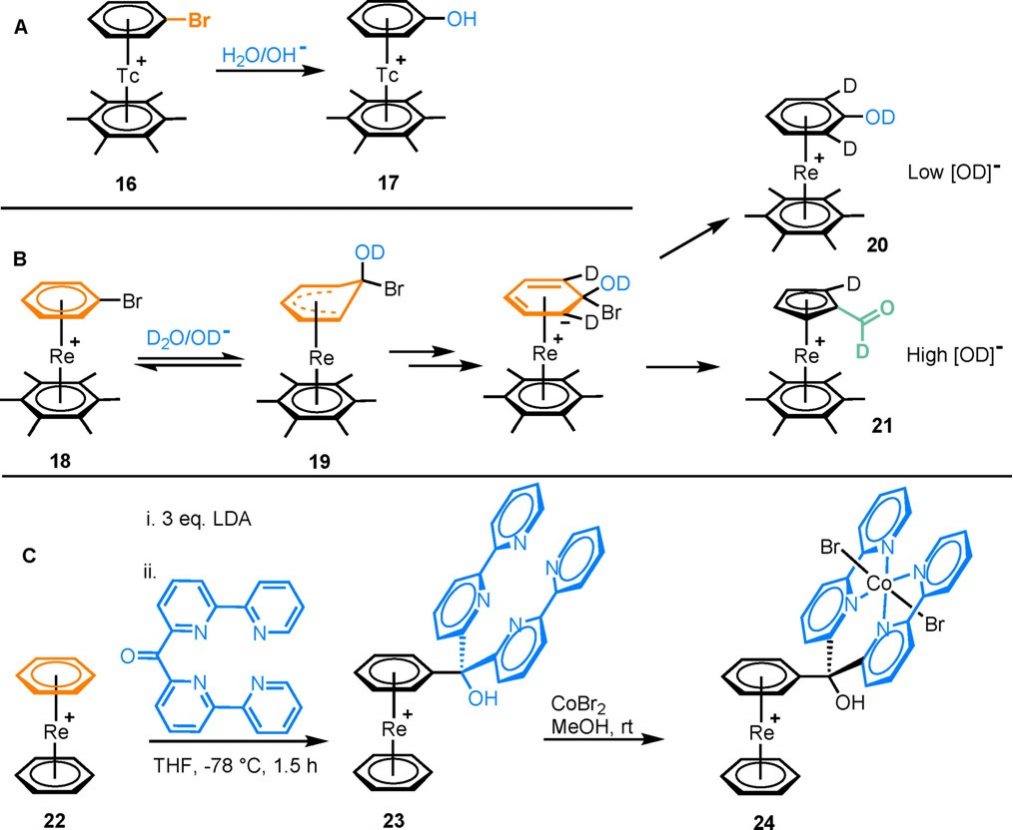
A) S_N_Ar hydroxylation of [Tc(η^6^‐C_6_H_5_Br)(η^6^‐C_6_Me_6_)]^+^ B) Nucleophilic hydroxylation of [Re(η^6^‐C_6_H_5_Br)(η^6^‐C_6_Me_6_)]^+^ followed by either formation of the S_N_Ar product or ring contraction, depending on base concentration. C) Functionalisation of benzene in [Re(η^6^‐C_6_H_6_)_2_]^+^ followed by synthesis of a bimetallic Re‐Co catalyst.

Unlike Mn complexes, there are very few examples of π‐arene complexes of technetium, although sandwich complexes of radioactive ^99m^Tc have been studied for their activity in biomimetic imaging.^[25]^ In a rare example by Alberto and co‐workers, an S_N_Ar hydroxylation of the complex [Tc(η^6^‐C_6_H_5_Br)(η^6^‐C_6_Me_6_)]^+^ (**16**) was described (Figure 3 A), forming η^6^‐phenol complex **17**.^[26]^ The corresponding rhenium complex (**18**) was found to undergo a ring contraction giving **21** (Figure 3 B) rather than the substitution product (**20**).^[26]^ Based on positions of H/D exchange in a deuterium experiment, a mechanism was proposed in which initially a nucleophilic attack of DO^−^ takes place to form the Meisenheimer intermediate **19**. H/D exchange was observed in this intermediate. An S_N_Ar reaction leading to complex **20** is promoted at lower deuteroxide concentrations, while ring contraction to form complex **21** is the favoured pathway when a larger excess of deuteroxide is present (Figure 3 B).

In a more recent study, the complex [Re(η^6^‐C_6_H_6_)_2_]^+^ (**22**) was functionalised with a polypyridyl group to form complex **23** (Figure 3 C).^[27]^ Coordination of a catalytically active Co^II^ centre to the polypyridyl moiety gave bimetallic complex **24**, which was active in the photocatalytic reduction of protons to H_2_ gas. The presence of the Re sandwich complex moiety in **24** adds structural support and flexibility to the catalytic species, as well as increasing aqueous solubility of the conjugate and adding resistance to deactivating redox processes.

### Iron complexes

**Figure 4:**
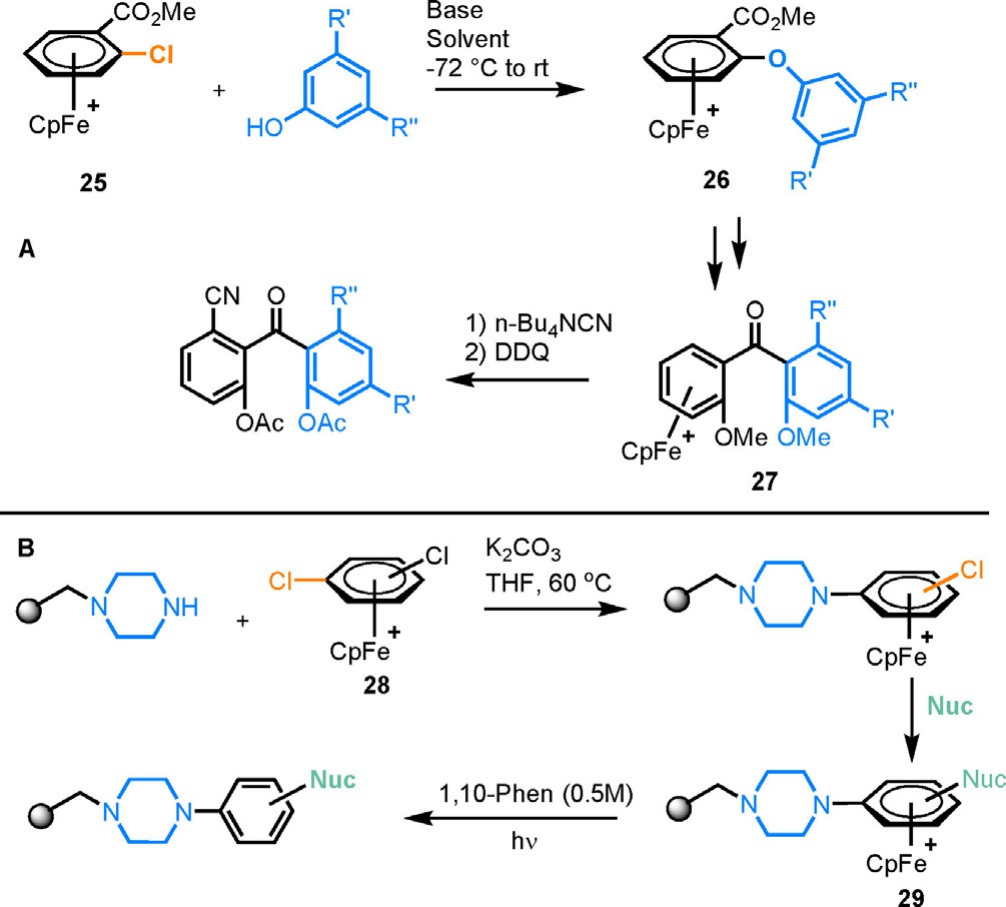
A) Fe‐mediated synthesis of a series of benzophenone derivatives. B) Fe‐mediated S_N_Ar on a solid‐phase support, with subsequent photolysis.

Arene π‐complexes of iron were extensively researched in the late 20th Century. Some key studies conducted by Pearson and Shin included the synthesis of cyclic peptides via S_N_Ar^[28]^ and the formation of aryl ethers from coordinated 1,3‐dichlorobenzene.^[29]^ Woodgate also used S_N_Ar reactions of coordinated arenes in the synthesis of dibenzo[*b*,*e*][1,4]dioxin derivatives[^30^, ^31^] and Astruc showed Fe‐mediated dendrimer synthesis, exploiting the increased acidity of benzyl protons on η^6^‐coordination.^[32]^ More recently, examples involving the use of iron have become scarce, due to the low stability of these complexes relative to Ru analogues. One of few examples is an S_N_Ar‐based approach to synthesise a series of unsymmetrically substituted sterically congested benzophenones (Figure 4 A).^[33]^ In a rare example of heterogeneous reactions involving π‐arene metal complexes, a piperazine nucleophile was tethered to a solid surface before S_N_Ar reactions with a range of [(η^6^‐chloroarene)FeCp]^+^ complexes were performed (Figure 4 B). The resultant immobilised Fe complexes were subjected to irradiation in the presence of phenanthroline to liberate the Fe‐arene bond and leave the aniline derivatives tethered to the solid surface.^[34]^

### Ruthenium complexes

Since the turn of the century, ruthenium has gathered significant traction in its activation of arenes through π‐coordination. This is due to the mild methods of complexation and demetallation, as well as high complex stability.

**Figure 5:**
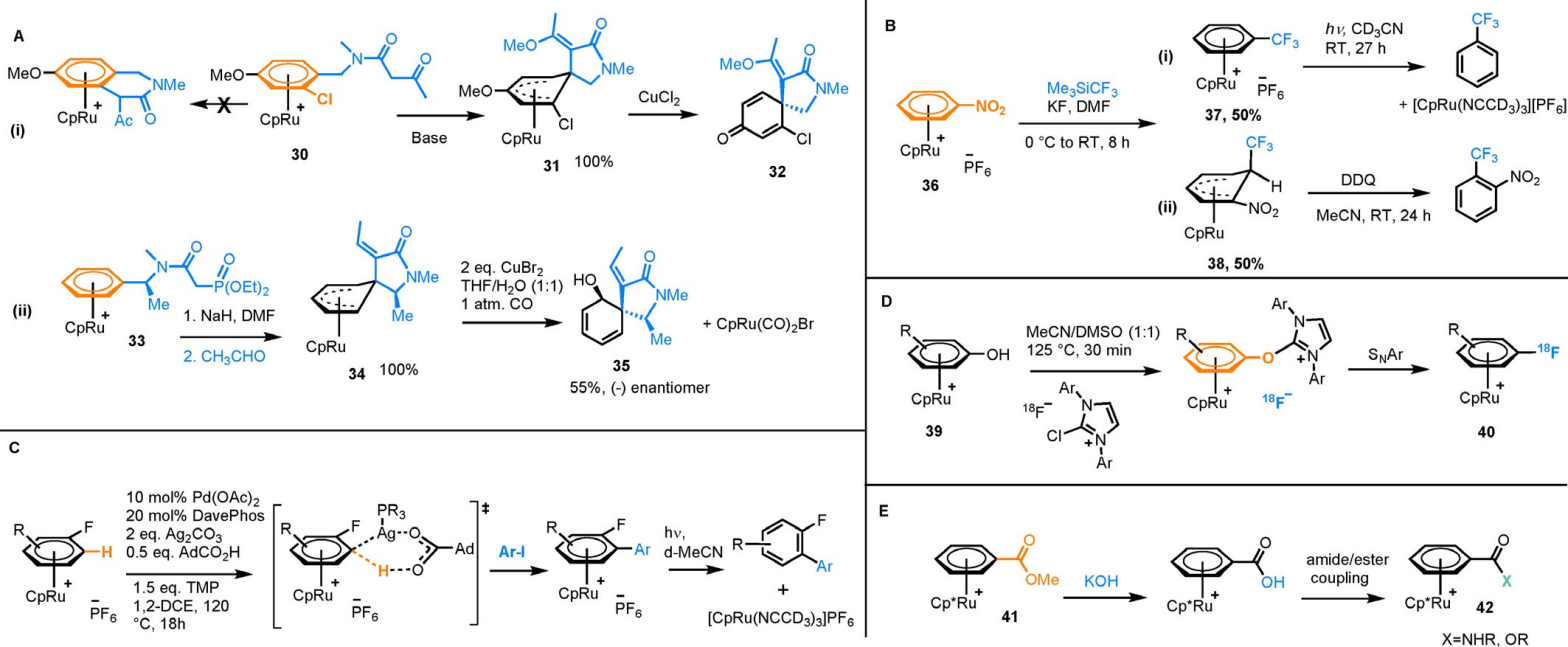
A) Intramolecular nucleophilic attack to form a spirolactam, and diastereoselective synthesis of a spirolactam derivative via a Ru‐coordinated Meisenheimer intermediate. B) Reaction of [(η^6^‐nitrobenzene)RuCp][PF_6_] with Me_3_SiCF_3_, followed by photolysis of the S_N_Ar product or oxidative rearomatisation to form free 1‐nitro‐2‐(trifluoromethyl)benzene. C) Aromatic C−H arylation mediated by a bimetallic Ag^I^/Pd^0^ catalysis. D) Ru‐facilitated deoxyfluorination procedure used for radiolabelling arenes with the isotope ^18^F. E) Ester hydrolysis of an η^6^‐coordinated benzyl ester.

In the early 2000s, Pigge and co‐workers produced a series of studies, in which spirolactams were synthesised via an intramolecular nucleophilic attack of a 1,3‐dicarbonyl enolate at the coordinated arene ring in complex **30** (Figure 5Ai).[^35^, ^36^, ^37^, ^38^] The nucleophilic attack occurred exclusively to form a five‐membered spirolactam core. Subsequent alkylation gave **31**, from which demetallation gave the corresponding unbound spirolactam products (**32**). In a follow‐up study, stereoselective spirolactam synthesis was achieved in a two‐step process involving cyclisation of **33**, followed by an intramolecular Horner–Wadsworth–Emmons (HWE) olefination to give complex **34** followed by demetallation to yield enantiopure spirolactam **35** (Figure 5Aii).^[37]^ This reaction demonstrates how stereocontrol is imparted by the presence of the activating metal complex. More recently, further novel polycyclic arene complexes of Ru were synthesised using the Morita–Baylis–Hillman reaction of phosphines.^[39]^

In recent years, work from our laboratory has focussed on π‐arene ruthenium reactivity. We developed a trifluoromethylation of a series of nitrobenzene derivatives (**36**, Figure 5 B), using Me_3_SiCF_3_.^[40]^ Two products were separated from the reaction in a 1:1 ratio: an S_N_Ar product **37**, which undergoes photolysis to release the free arene and a Meisenheimer complex **38** from nucleophilic attack of the CF_3_ group at an aromatic C−H *ortho* to the C−NO_2_ bond. The latter product was treated with DDQ, which causes oxidative rearomatisation and demetallation to give 1‐nitro‐2‐(trifluoromethyl)‐benzene. A second example developed from our laboratories is a Pd/Ag‐mediated arene C−H activation/arylation. Building on pioneering work from Larrosa,^[41]^ who demonstrated C−H activation in [(η^6^‐arene)Cr(CO)_3_] complexes, we showed the potential for C−H activation in [(η^6^‐arene)RuCp]^+^ complexes (Figure 5 C).^[42]^ Two catalytic metals (Pd and Ag) are required, as the mechanism involves an initial Ag‐mediated concerted metalation deprotonation (CMD) C−H activation step, before undergoing transmetalation to form the activated aryl C−Pd bond. As with our previous trifluoromethylation S_N_Ar process, liberation of the functionalised arenes from their Ru complexes was achieved by photolysis in deuterated acetonitrile, which also gave quantitative [CpRu(NCCD_3_)_3_]^+^ by‐product, indicating recovery of the starting Ru complex is feasible.

Two practical applications of Ru π‐arene complexes include the synthesis of radiolabelled aryl fluorides from aryl alcohol^[43]^ (Figure 5 D) and the ester hydrolysis of lignin model compounds.^[44]^ Furthermore, Ru‐promoted hydrolysis of ester groups was also used to synthesise new cytotoxic organometallics (Figure 5 E).^[45]^ Ester hydrolysis in the complex [(η^6^‐C_6_H_5_CO_2_Me)RuCp*][BF_4_] (**41**) was followed by the synthesis of a small library of ester and amide derivatives (**42**), several of which showed promising toxicity against various cancer cell lines.

### Rhodium and iridium complexes

**Figure 6:**
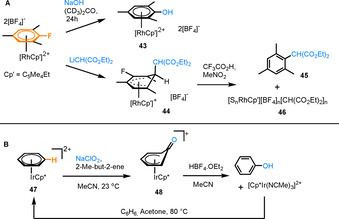
A) A series of reactions of [Cp'Rh(η^6^‐1‐fluoro‐2,4,6‐methyl)benzene][BF_4_]_2_ with various nucleophiles. B) Ir^III^‐mediated benzene C−H hydroxylation.

Despite the prevalence of π‐arene complexes of rhodium and iridium,[^46^, ^47^] there are a very limited number of examples where the arene is undergoing transformation while coordinated to the metal. One study demonstrates the reactivity of a series of [(η^6^‐arylfluoride)RhCp’]^2+^ complexes (Cp’=tetramethyl(ethyl) cyclopentadienyl). Reactivity of these complexes is highly dependent on the nature of the nucleophile (Figure 6 A).^[48]^ Hydroxide targets the polarised C−F bond, leading to an S_N_Ar process to give complex **43** (Figure 6 A), while alkyllithium nucleophiles attack one of the unsubstituted aryl C atoms. Reaction with LiCH(CO_2_Et)_2_ results in Meisenheimer complex **44**, which can undergo in situ oxidative rearomatisation to the free arene **45**, using trifluoroacetic acid (TFA) and nitromethane. It is worth noting here that a Rh complex **46** was also observed following the reaction, emphasising a non‐destructive demetallation process.

A single example of the reactivity of iridium π‐arene complexes (Figure 6 B) involves oxidation of [Cp*Ir(η^6^‐C_6_H_6_)]^2+^ (**47**) to form the corresponding η^5^‐cyclohexadienyl oxide (**48**).^[49]^ Acidification with HBF_4_⋅OEt_2_ in acetonitrile liberates phenol and [Cp*Ir(NCMe)_3_]^2+^ with a yield of 75 %. The overall process is catalytic in Ir, as **47** can be regenerated from the Ir product, however, the conditions for oxidation and decomplexation are incompatible and the reaction requires isolation at each stage.

### Perspectives

The reactions described in this section demonstrate the wide range of chemical reactivity of arenes bound to various transition metals and highlights their potential applications in a key area of industrial and pharmaceutical chemistry. Coordination to the metal centre enhances the reactivity of the arene and can control the regiochemistry of the reaction. Furthermore, as the metal coordinates to one face of the arene, complexation can allow for control of stereochemistry and enantioselective syntheses. This stereocontrol has been used in the production of pharmaceutically relevant target compounds, such as the stemofurans. As a choice of activating metal, [Mn(CO)_3_]^+^ fragments are commonly used, due to their ease of synthesis and manipulation. However, the oxidative techniques used to recover the arene product lead to loss of the Mn complexes. To develop more efficient and greener processes it would be desirable to develop reactions in which the Mn^I^ activating metal could be recovered or reused. As an alternative, Ru has become more prevalent as an activating metal, due to the ease of synthesis of their complexes and the ability to remove the reaction product by photolysis, regenerating the activating Ru fragment. For this method of aromatic activation to become competitive with other synthetic methodologies, efficient recovery of the metal is paramount. While homogeneous reactivity of activating π‐arene complexes is common, only a single heterogeneous reaction is known. There is great potential here for future studies on reactions in which the activating metal is tethered to a solid support to allow ease of purification and potentially access to flow systems for rapid and efficient synthesis. A key limitation to the application of these reactions is the need for stoichiometric activating metal. This negative effect can be offset by the ability to recycle the activating metal, however, a more desirable approach is to develop reactions that are catalytic in metal and proceed through transient formation of the active π‐arene complex. In the next section, we give an overview of reactions that are catalytic in the activating metal.

## Catalytic Transformations via Transient η6‐Coordination

One challenge to developing catalytic reactions involving π‐arene intermediates is finding conditions that are compatible with both the transformation step and arene exchange. A balance between arene reactivity and arene exchange ability is necessary, because a stronger arene‐metal interaction typically leads to a greater reactivity of the ring, but disfavours arene exchange. The factors that affect arene exchange have been reviewed elsewhere.[^3^, ^6^] Despite the difficulty in achieving this balance, there are several cases published, mostly in the past 10 years, where the arene transformation is catalytic in the activating metal.

### Reactions catalytic in ruthenium

**Figure 7:**
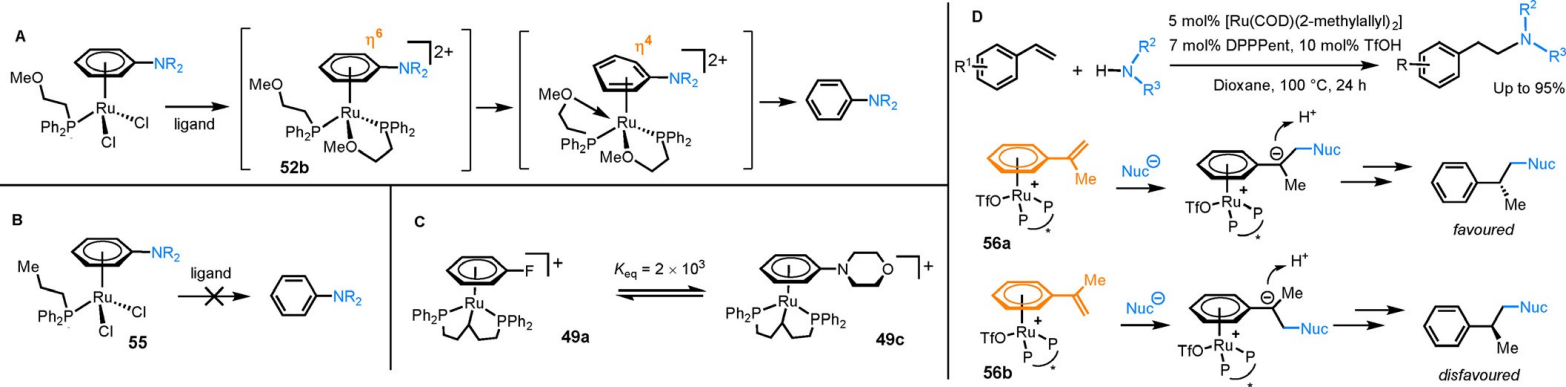
A) Accelerated arene exchange promoted by transient coordination of a hemilabile methoxy phosphine ligand during rate limiting η^6^ to η^4^ step. B) No arene exchange shown for analogous ligand without coordinating methoxy group. C) Equilibrium constant between Ru complexes containing reactant and product capping arenes. D) Hydroamination catalysed by transient Ru coordination and the introduction of enantioselectivity by using a chiral bisphosphine ligand ($\hat{PP}$ *=(*S*)‐xylylBINAP).

The most common class of catalytic reactions proceeding via η^6^‐arene intermediates are S_N_Ar reactions with catalytic Ru.^[50]^ The first example came from Shibata on the amination of unactivated fluoroarenes, proceeding via Ru^II^ intermediates **49 a** and **49 b** (Table 1, Entry 1).^[51]^ Reaction conditions involved a Ru cyclooctadiene catalyst, phosphine DPPPent ligand and additives TfOH and Et_3_SiH. Recently, Mueller and Schley carried out a detailed mechanistic study on the reaction between fluorobenzene and morpholine, under these catalytic conditions.^[52]^ Product inhibition was observed and quantified, as the *N*‐phenylmorpholine reaction product binds much more strongly to Ru than fluorobenzene (*K*_eq_=2×10^3^ for the equilibrium between the fluoro, **49 a**, and morpholino species, **49 c**), limiting the rate of arene exchange (Figure 7 C). Furthermore, the resting state of catalysis was found to be a previously unknown Ru‐hydride species **50 b** (Table 1, Entry 2. X=morpholino). The formation of this species reveals the role of additives Et_3_N and Et_3_SiH, which drive the formation of the more active species **50 b** from **49 b**. Indeed an alternative catalyst, in which the DPPPent ligand in **50 b** is replaced by two PPh_3_ ligands gives comparable yields (72 % versus >99 % for **50 b**). In follow up work by Shibata, a bench stable catalyst [Ru(η^6^‐C_6_H_6_)Cl_2_]_2_ and an alternative phosphine ligand led to improvements in reaction efficiency, with yields >80 % (Table 1, Entry 3).^[53]^ This reaction proceeded via intermediates **51 a** and **51 b**.

**Table 1:** Catalytic S_N_Ar proceeding through π‐arene intermediates.

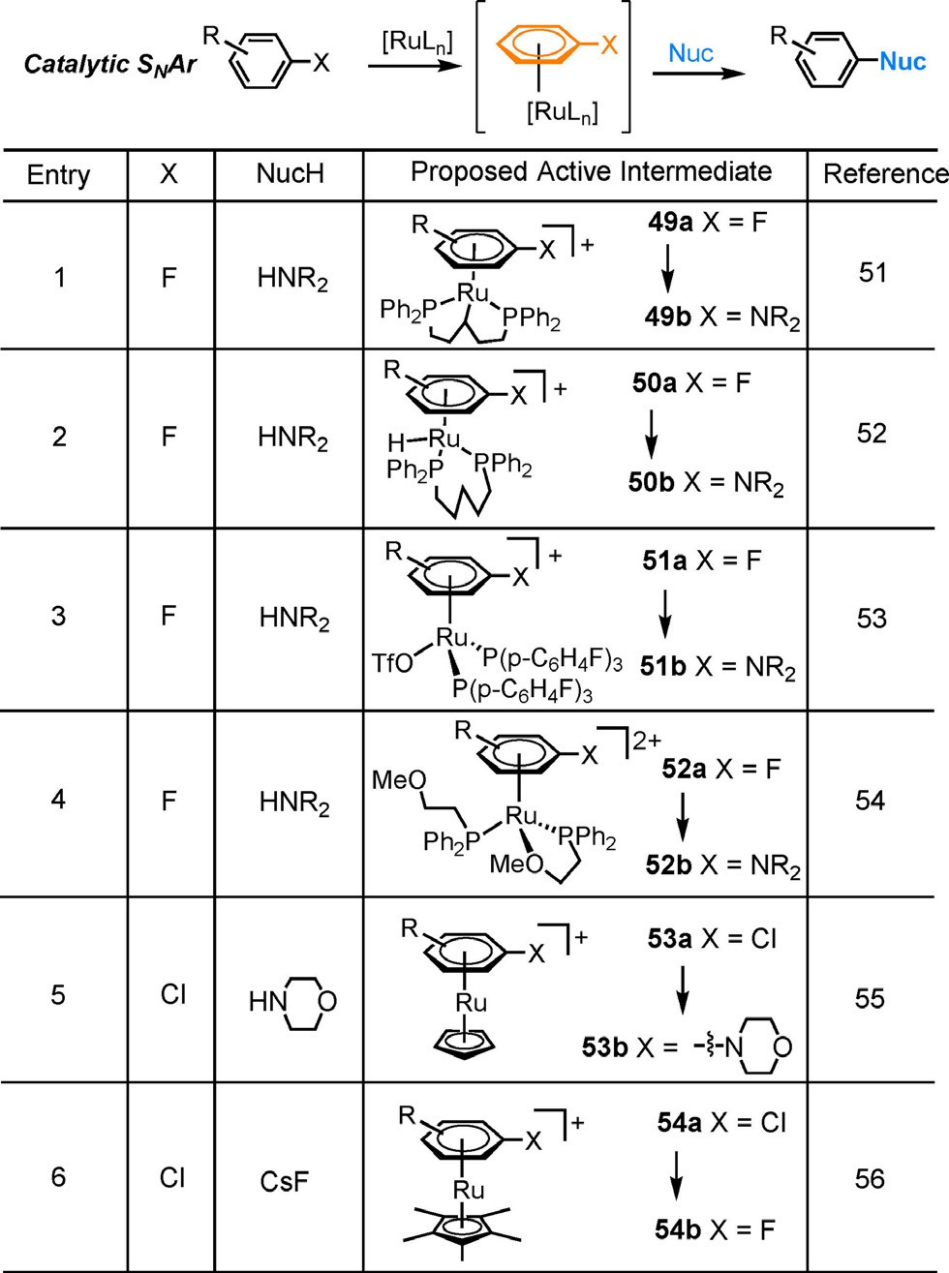

Entry 1 conditions: Arene (5 equiv), NucH (1 equiv), [Ru(COD)(2‐methylallyl)_2_] (5 mol %), DPPPent (7 mol %), TfOH (10 mol %), Et_3_SiH (1 equiv), Et_3_N (1 equiv), dioxane, 100 °C. Entry 2 conditions: Arene (5 equiv), NucH (1 equiv), **50 b** (X=morpholino, 5 mol %), dioxane, 100 °C. Entry 3 conditions: Arene (5 equiv), NucH (1 equiv), [Ru(η^6^‐C_6_H_6_)Cl_2_]_2_ (2.5 mol %), P(*p*‐C_6_H_4_F)_3_ (24 mol %), AgOTf (21 mol %), 1,4‐Dioxane, 100 °C. Entry 4 conditions: Arene (1 equiv), NucH (3 equiv), [Ru(η^6^‐C_6_H_6_)Cl_2_]_2_ (2.5 mol %), PPh_2_(C_2_H_4_OMe) (10 mol %), AgPF_6_ (10.5 mol %), THF, 120 °C. Entry 5 conditions: Arene (1 equiv), NucH (3 equiv), [CpRu(η^6^‐*p*‐cymene)][PF_6_] (10 mol %), 1‐octanol, 180 °C. Entry 6 conditions: Arene (excess), CsF (1 equiv), [Cp*Ru(η^6^‐naphthalene)][BF_4_] (2 mol %), neat arene, 180 °C.

In 2020, Shi and co‐workers presented a similar procedure for ruthenium catalysed S_N_Ar coupling of fluoroarenes with amines (Table 1, Entry 4), proceeding via intermediates **52 a** and **52 b**.^[54]^ By using a hemilabile phosphine ligand, a balance between reactivity and arene exchange was achieved and the reaction proceeded to excellent yields for a range of aryl fluorides and amines under mild reaction conditions. Key to this success was the proposed catalytic intermediates, **52 a** and **52 b**, which incorporate two phosphine ligands: one bidentate and the other monodentate. Rapid S_N_Ar is followed by arene exchange, which is accelerated through transient coordination of the second hemilabile phosphine ligand (Figure 7 A). As evidence for this process, a related phosphine ligand without the second coordinating group showed no release of the reaction product from complex **55** (Figure 7 B).

The previous examples of catalytic S_N_Ar are limited to fluoroarenes, with no reactivity shown for aryl chlorides or bromides. Successful reaction procedures using chloroarenes have been developed more recently. The first example is the catalytic S_N_Ar of aryl chlorides shown by our laboratory in 2015 (Table 1 Entry 5).^[55]^ Using 10 mol % of the precatalyst [CpRu(η^6^‐*p*‐cymene)][PF_6_], *p*‐chlorotoluene was coupled with morpholine in 90 % yield. From spectroscopic data, we inferred complex **53 a** as the active intermediate. In a related study, Grushin achieved a catalytic fluorination of aryl chlorides via S_N_Ar (Table 1, Entry 6).^[56]^ Using the pre‐catalyst [Cp*Ru(η^6^‐naphthalene)][BF_4_] and CsF as the nucleophilic fluoride source, the reaction proceeded at 140 °C in dry DMF, via intermediates **54 a** and **54 b**, with a catalytic turnover number (TON) of 4.3 after 24 hours. Compared with the previous example, this reaction works at a lower temperature, which is potentially due to the more electron‐rich Cp* facilitating arene exchange better than Cp. When the reaction was performed in neat chlorobenzene at 180 °C, an improved TON of 8.5 was observed.

Catalytic S_N_Ar reactions are all activated by the increase in electrophilicity of the Ru‐bound aromatic ring. This enhancement of reactivity also extends beyond the coordinated ring to more distal positions. The earliest example of catalytic activation via this route was the Ru‐catalysed anti‐Markovnikov hydroamination of styrene derivatives with secondary amines, reported by Hartwig et al. in 2004 (Figure 7 D).^[57]^ In this study, the hydroamination was facilitated by the stabilisation of the Ru‐bound intermediate that places negative charge in the α‐position of coordinated styrene. Conjugation with the coordinated arene provides this stabilisation and the resultant optimised yields were up to 95 %. In a related study, enantioselective addition to α‐methylstyrene was achieved using a chiral bisphosphine ligand.^[58]^ Chirality in the ligand renders complexes **56 a** and **56 b** diastereomeric. After nucleophilic attack, protonation occurs on the less hindered face away from the Ru, hence a preference for one diastereomer ultimately leads to enantioselectivity in the final uncoordinated product.

**Figure 8:**
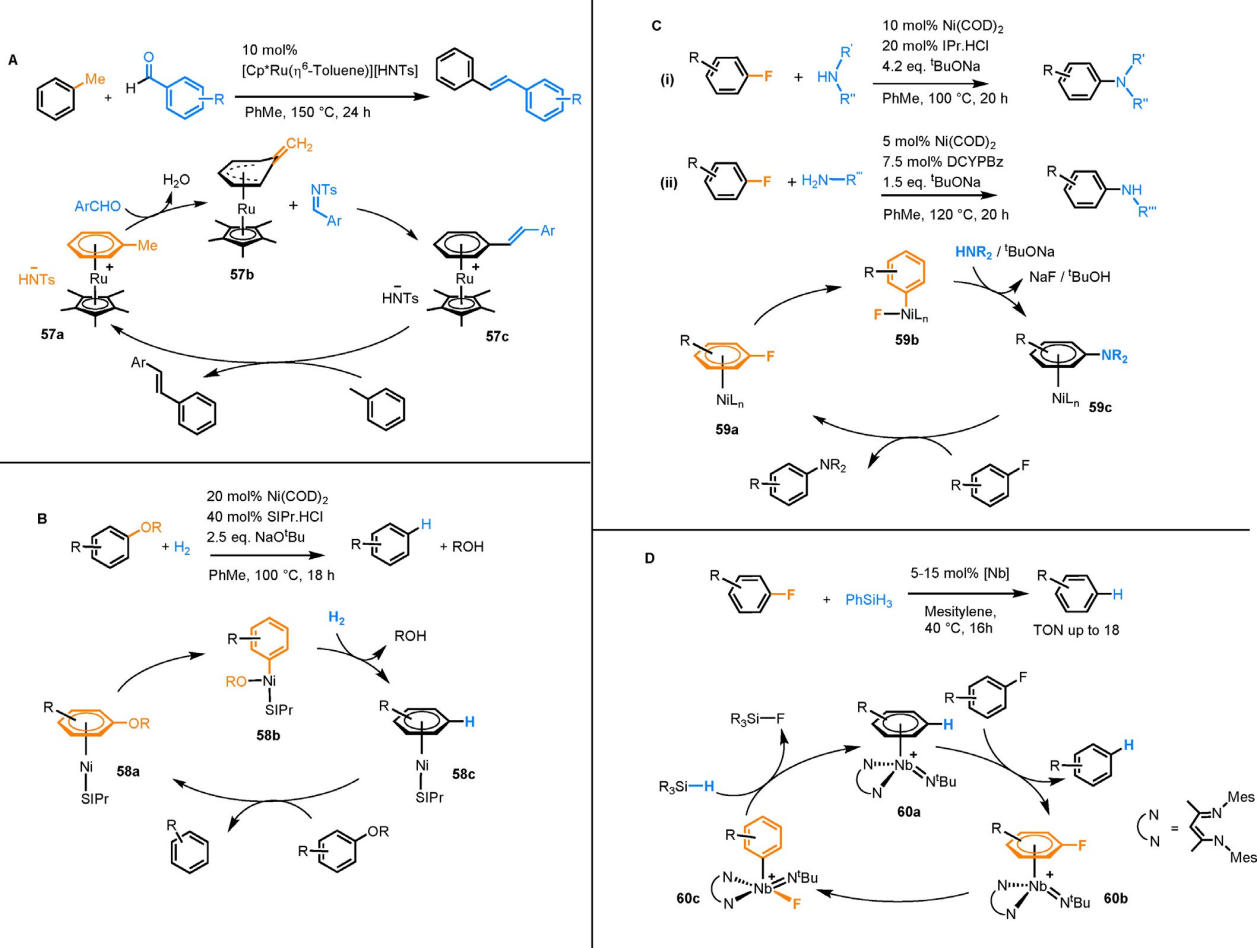
A) Ru‐catalysed dehydrative condensation of styrene derivatives. B) Ni^0^‐catalysed hydrogenation of aryl ethers, C) Ni^0^‐catalysed amination of aryl fluorides and D) Nb‐catalysed hydrodefluorination of a series of aryl fluorides.

A final example of catalysis via transient Ru π‐arene complexes exploits the increased acidity of benzylic protons of coordinated arenes.^[59]^ The production of *trans*‐stilbene derivatives was shown via a dehydrative condensation of benzylic C−H bonds with aromatic aldehydes (Figure 8 A).^[60]^ Using 10 mol % of the catalyst [Cp*Ru(η^6^‐toluene)][HNTs], toluene was coupled with a series of aromatic aldehydes at 150 °C in yields of up to 95 %. The key intermediate in the mechanism is **57b**, in which deprotonation at the benzylic position of toluene is stabilised by Ru coordination. As shown in Figure 8 A, this intermediate goes on to react with benzaldehyde, which itself is activated to an imine by the Ru complex counterion [HNTs]^−^. The organocatalytic behaviour of this ion was found to be crucial in the reaction, as other counter ions (PF_6_^−^, TfO^−^, Cl^−^) resulted in no stilbene formation. During catalyst screening, the [CpRu]^+^ system was found to be less active than the [Cp*Ru]^+^ fragment, which implies that arene exchange is rate‐determining, as a more electron rich metal centre is expected to lead to weaker η^6^‐arene coordination.

### Reactions catalytic in nickel

Beyond reactions catalytic in Ru, several examples have been reported in which π‐arene Ni intermediates are implicated. In a series of papers Hartwig reported the hydrogenation of aryl ethers with H_2_ to yield aryl alcohols and unsubstituted arenes (Figure 8 B), using catalytic Ni(COD)_2_ and an *N*‐heterocyclic carbene ligand.[^61^, ^62^] In the reaction, the aryl ether coordinates to a Ni^0^ centre via η^6^‐coordination to form intermediate **58 a**. The π‐coordination activates the aryl ether towards an oxidative addition to the Ni centre (**58 b**) (likely via an η^2^ intermediate^[63]^), before reaction with H_2_ leads to release of the aryl alcohol and regeneration of an η^6^‐coordinated unsubstituted arene (**58 c**). Finally, arene exchange with the more electron rich aryl ether starting material completes the catalytic cycle.^[62]^

Using very similar catalytic conditions, aryl fluorides were coupled with secondary amines to give substituted anilines (Figure 8 C).^[64]^ While no direct evidence for η^6^‐coordination was provided, an oxidative addition pathway was proposed and based on Hartwig's subsequent work, a π‐arene intermediate preceding oxidative addition seems highly likely. The scope of this reaction was extended to include primary amines in a study led by Iwai and Sawamura.^[65]^ Again, a Ni^0^‐based catalyst was employed, but bulky bis(phosphine) ligands were used instead of *N*‐heterocyclic carbenes, giving selectivity for formation of secondary over tertiary amines.

### Reactions catalytic in niobium

A single example has been presented in the literature in which Nb π‐arene intermediates have been identified as key reaction intermediates.^[66]^ Aryl fluorides undergo arene exchange with Nb^III^ complex **60 a** to give **60 b**, which undergoes oxidative addition to form aryl‐Nb^V^ intermediate **60 c**. This species reductively eliminates in the presence of stoichiometric phenyl silane to give an overall catalytic hydrodefluorination (Figure 8 D).

### Perspectives

**Figure 9:**
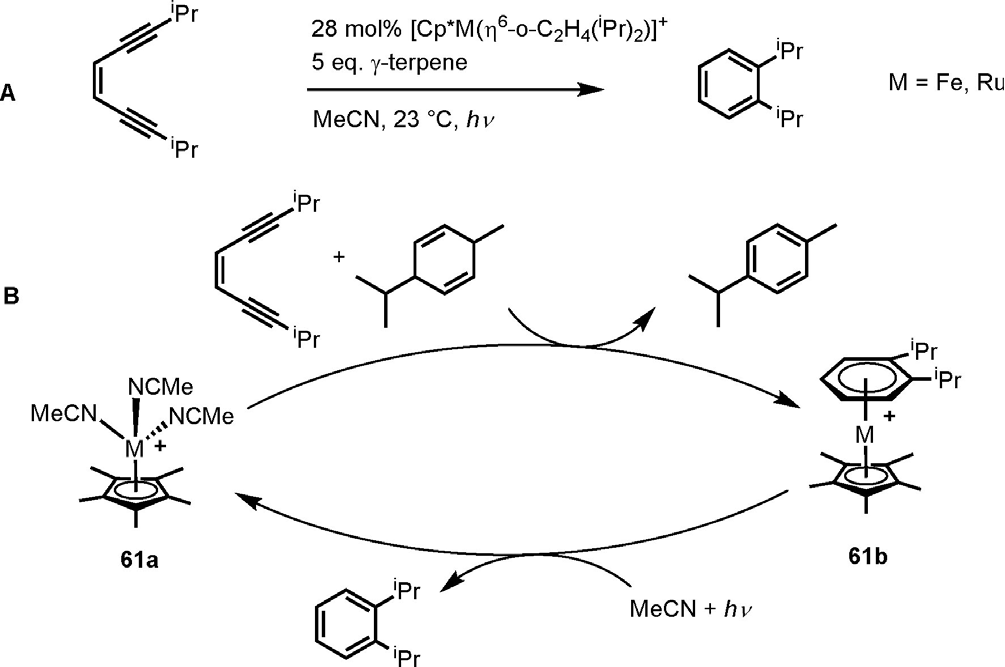
A) Bergman cycloaromatisation of an enediyne catalysed by Fe or Ru and irradiation with light. B) Proposed mechanism of photoactivated catalytic cycloaromatisation.

Whilst progress has been made over the past decade in developing catalysis via π‐arene intermediates, limitations still remain, with most reactions suffering from low TONs and poor scope of reactivity. These factors must be improved upon if these reactions are to compete with catalytic transformations used in industrial processes. As previously discussed, factors that promote reactivity of a ring typically inhibit its ability to undergo arene exchange and mastering this fine balance is key to improving the TON of a catalyst. Another factor complicating the arene exchange step is that often displacement of the product aromatic with the starting material is disfavoured by their relative thermodynamic stabilities, particularly where electron rich nucleophiles are involved. With both issues in mind, careful consideration of substrates is necessary or methods to trigger arene exchange in stable complexes are required. The field of photocatalysis has grown rapidly over recent years, due to advances in both theoretical understanding and practical instrumentation. It is well known that certain π‐arene metal complexes are susceptible to photoactivated destabilisation of the metal arene bond, therefore catalytic reactions in which light is used to trigger arene exchange should be entirely feasible. Despite this, there are almost no examples of this type of photocatalytic reactivity. A single reaction is known in which a Ru‐ or Fe‐catalysed cycloaromatisation of an enediyne with γ‐terpinene as H source is shown to proceed under irradiation (Figure 9).^[67]^ The proposed catalytic mechanism (Figure 9 B) consists of a cycloaromatisation to form an η^6^‐coordinated arene complex, **61 b**, then photolysis to give the free arene and the complex [Cp*M(NCMe)_3_]^+^ (**61 a**), restarting the catalytic cycle. It is likely that the emergence of more photocatalytic protocols can pave the way towards solutions surrounding arene exchange, and developments here must be considered a high priority in the coming decade.

## Conclusions and Outlook

This review was written with the objective of giving a comprehensive summary of recent advances in the reactivity of capping aromatics in π‐arene metal complexes and transformations that are catalytic in metal. Despite the fact that this field has been established since the 1950s, many significant developments have been made in the past 10—15 years. Many new reactions that are stoichiometric in metal, both on the arene ring and its periphery have been reported, which highlight the significant change in reactivity of an arene upon coordination and the potential for stereochemical control. Furthermore, by combining the increased reactivity of η^6^‐coordinated arenes with conditions that allow arene exchange, several efficient catalytic protocols have been developed.

Looking forwards, several key milestones are still to be met in this field. Firstly, reactions that are stoichiometric in metal would ideally be adapted such that the metal centre can either operate catalytically or allow for simple recovery of the activating metal. Photolytic liberation of the capping arene and/or the use of heterogeneous systems are the most promising methods to achieve recovery of the activating metal, but only Ru complexes currently allow for simple liberation of the coordinated arene via these methods. Whilst the development of catalytic reactions has been a major breakthrough in this field, more needs to be achieved to be competitive with industrial processes. In‐depth mechanistic studies that help to identify active catalytic species and rate limiting reaction steps are crucial to realise this ambition. Focus on developing photocatalytic reactions also seems an important route forward. Particularly, as advances are made in both theoretical and practical photochemistry, it is key for the field of π‐arene metal complexes to take advantage of these developments. Overall, this field has a bright outlook and we look forward to the next decade of developments.

## Conflict of interest

The authors declare no conflict of interest.

## Biographical Information

*Luke Williams received his MChem degree from the University of Warwick in 2018. Currently, he is a third‐year PhD student at the University of Durham. Under the supervision of Dr. James Walton, he is exploring the chemistry of ruthenium sandwich complexes and their applicability towards the catalysis of aromatic transformations*.

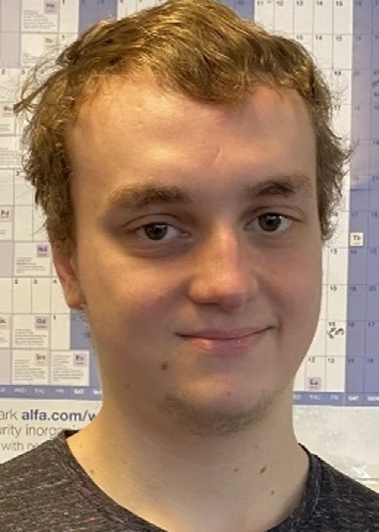

## Biographical Information

*Yunas Bhonoah graduated with a MSci in Chemistry with Medicinal Chemistry from Imperial College London in 2005. He stayed at Imperial for postgraduate studies under the supervision of Prof. Alan Armstrong and received his PhD in 2009. He then joined Syngenta, UK, where he is currently Principal Research Chemist working in crop protection research*.

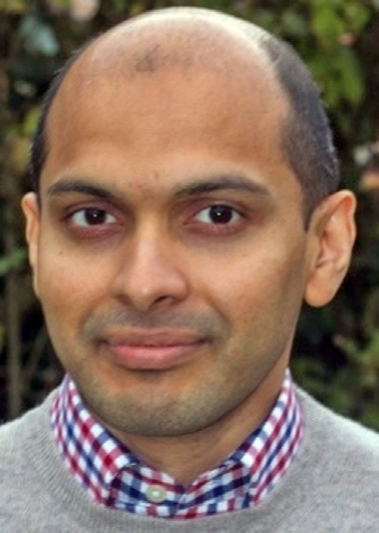

## Biographical Information

*Luke A. Wilkinson obtained his MChem from the University of East Anglia and then undertook a PhD with Prof. Nathan Patmore at the Universities of Sheffield and Huddersfield, exploring novel charge transfer mechanisms between dimetal paddlewheel complexes. He then took up a PDRA position at Durham University with Dr. James Walton on the reactivity of (η^6^‐arene)M complexes, followed by a PDRA at Imperial College with Prof. Nicholas Long on the synthesis of molecular electronic components for thermoelectric applications. Luke is now a Leverhulme Trust Early Career Research Fellow at the University of York where he is constructing dimolybdenum–porphyrin dyads for solar harvesting applications*.

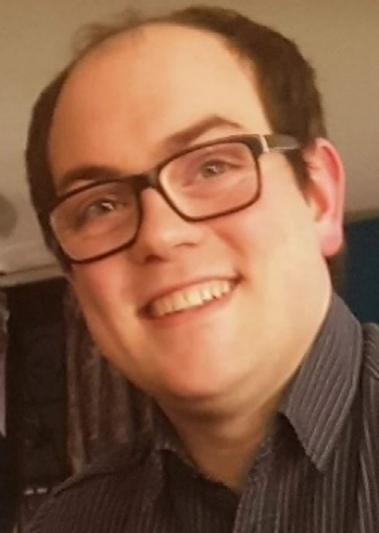

## Biographical Information

*James W. Walton graduated with an MChem degree from Durham University in 2008. He then completed a PhD on Emissive Lanthanide Complexes under the supervision of Prof. David Parker at Durham University. He completed postdoctoral work with Prof. Jonathan Williams at the University of Bath on ruthenium organometallic complexes in catalysis. Since 2014, he has been an Assistant Professor in Inorganic Chemistry at Durham University, where his research focuses on organometallic complexes in catalysis and as therapeutic agents*.